# Clozapine-Encapsulated Binary Mixed Micelles in Thermosensitive Sol–Gels for Intranasal Administration

**DOI:** 10.3390/gels8010038

**Published:** 2022-01-05

**Authors:** Madeleine S. A. Tan, Preeti Pandey, James R. Falconer, Dan J. Siskind, Alexandra Balmanno, Harendra S. Parekh

**Affiliations:** 1 School of Pharmacy, The University of Queensland, 20 Cornwall Street, Woolloongabba, QLD 4102, Australia; madeleine.tan1@uq.edu.au (M.S.A.T.); j.falconer@uq.edu.au (J.R.F.); 2 School of Medicine, The University of Queensland, 20 Weightman Street, Herston, QLD 4006, Australia; d.siskind@uq.edu.au; 3 Metro South Addiction and Mental Health Service, Level 2 Mental Health, Woolloongabba Community Health Centre, 228 Logan Road, Woolloongabba, QLD 4102, Australia; 4 School of Veterinary Science, The University of Queensland, 5391 Warrego Highway, Gatton, QLD 4343, Australia; a.lonergan1@uq.edu.au

**Keywords:** antipsychotic, cardiomyopathy, clozapine, constipation, hydrogel, hypersalivation, metabolic syndrome, nose-to-brain delivery, poloxamer gel, polysorbates, postural hypotension, schizophrenia

## Abstract

(1) Background: Clozapine is the most effective antipsychotic. It is, however, associated with many adverse drug reactions. Nose-to-brain (N2B) delivery offers a promising approach. This study aims to develop clozapine-encapsulated thermosensitive sol–gels for N2B delivery. (2) Methods: Poloxamer 407 and hydroxypropyl methylcellulose were mixed and hydrated with water. Glycerin and carbopol solutions were added to the mixture and stirred overnight at 2–8 °C. Clozapine 0.1% *w*/*w* was stirred with polysorbate 20 (PS20) or polysorbate 80 (PS80) at RT (25 °C) before being added to the polymer solution. The final formulation was made to 10 g with water, stirred overnight at 2–8 °C and then adjusted to pH 5.5. (3) Results: Formulations F3 (3% PS20) and F4 (3% PS80) were selected for further evaluation, as their gelation temperatures were near 28 °C. The hydrodynamic particle diameter of clozapine was 18.7 ± 0.2 nm in F3 and 20.0 ± 0.4 nm in F4. The results show a crystallinity change in clozapine to amorphous. Drug release studies showed a 59.1 ± 3.0% (F3) and 53.1 ± 2.7% (F4) clozapine release after 72 h. Clozapine permeated after 8 h was 20.8 ± 3.0% (F3) and 17.8 ± 3.1% (F4). The drug deposition was higher with F4 (144.8 ± 1.4 µg/g) than F3 (110.7 ± 2.7 µg/g). Both sol–gels showed no phase separation after 3 months. (4) Conclusions: Binary PS80-P407 mixed micelles were more thermodynamically stable and rigid due to the higher synergism of both surfactants. However, binary mixed PS20-P407 micelles showed better drug permeation across the nasal mucosa tissue and may be a preferable carrier system for the intranasal administration of clozapine.

## 1. Introduction

Clozapine is the most effective antipsychotic for treatment-resistant schizophrenia [1]. It is, however, associated with many peripheral adverse drug reactions (ADRs), including cardiac (myocarditis, cardiomyopathy and postural hypotension), metabolic (metabolic syndrome, obesity and diabetes), hypersalivation and gastrointestinal (constipation and ileus) [2,3]. Nose-to-brain (N2B) drug delivery offers a promising approach to the delivery of antipsychotics to the central nervous system (CNS) in the management of schizophrenia. This route bypasses the blood–brain barrier (BBB) and hepatic first-pass metabolism, which may increase the CNS drug availability and reduce peripheral ADRs [4]. In order to deliver therapeutic doses of clozapine to the olfactory region, the antipsychotic drug needs to penetrate the hydrophilic mucus layer, while avoiding mucin adsorption, before permeating the olfactory membrane [5]. Due to the small volume of the nasal cavity, only small volumes (100–250 µL [6]) of therapeutic doses can be administered intranasally. It is, therefore, crucial for the antipsychotic drug to be solubilized before administration in order to increase the transmucosal nasal absorption and avoid the rapid mucociliary clearance from the nasal cavity [4].

Well-reported methods to enhance drug solubility include the use of surfactants and solubilizers [7,8,9]. Polysorbates are hydrophilic, non-ionic surfactants that are frequently used as an emulsifying and solubilizing agent in foods, cosmetics and pharmaceutical formulations [10]. Polysorbate 20 (PS20) and polysorbate 80 (PS80) are approved by the U.S. Food and Drug Administration (FDA) for intranasal administration, and are often used to solubilize hydrophobic drugs, such as clozapine. In recent years, poloxamer hydrogels have received interest as in situ drug delivery vehicles due to their thermoreversibility. Poloxamer 407 (P407) is among the most commonly used poloxamers, as it remains a solution at room temperature but forms an in situ gel at physiological temperatures and at low concentrations. It is a versatile excipient that is widely used in pharmaceutical formulations due to its low toxicity. Traditional surfactants have a low molecular weight, whereas P407 is made of triblock copolymers of poly(ethylene oxide)-*b*-poly(propylene oxide)-*b*-poly(ethylene oxide; PEO-PPO-PEO). The high hydrophobic PPO fraction of P407, along with the polymer molecular weight compared to other types of poloxamers, has the capacity to increase the drug loading of water-insoluble drugs through direct solubilization [11]. 

Designing the most suitable sol–gel formulation for the intranasal delivery of clozapine involves balancing a variety of parameters, including the micellarized drug, particle size, lipophilicity, formulation pH and molecular interactions between the drug and other excipients. Herein, we report on the development of clozapine-encapsulated binary polysorbate/poloxamer mixed micelles formulated into a thermosensitive sol–gel, intended for N2B delivery to reduce peripheral ADRs, increase CNS drug bioavailability and improve patient compliance. Accordingly, the aim of the present study was to investigate the feasibility of the optimized formulations to transport clozapine across nasal mucosa tissues in a controlled manner for a sustained therapeutic effect. 

## 2. Results and Discussion

### 2.1. Formulation Development and Optimization

Preliminary sol–gel formulations consisting of 13.5–16.5% *w*/*w* P407, without clozapine or additives, were prepared using Milli-Q water and evaluated for T_sol/gel_. As seen in Table 1, the T_sol/gel_ of the formulations increases as the concentration of P407 decreases. Sol–gels that demonstrated T_sol/gel_ of 28 °C (i.e., gelling starts at 28 °C) were selected so that the gelation phase is completed around the nasal temperature of 32–34 °C [12,13]. Therefore, sol–gels with 15.5% *w*/*w* P407 were selected for further investigations. 

**Figure 1:**
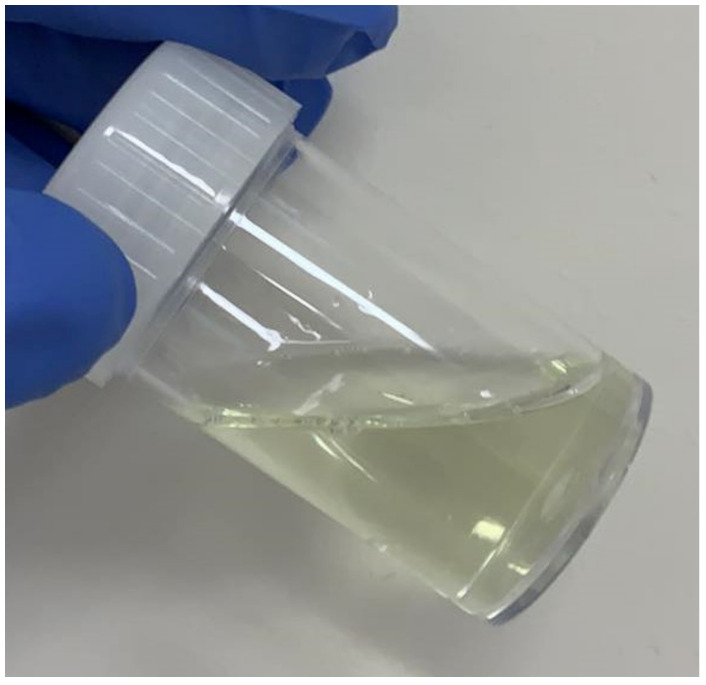
Binary PS20/P407 mixed micelles.

**Figure 2:**
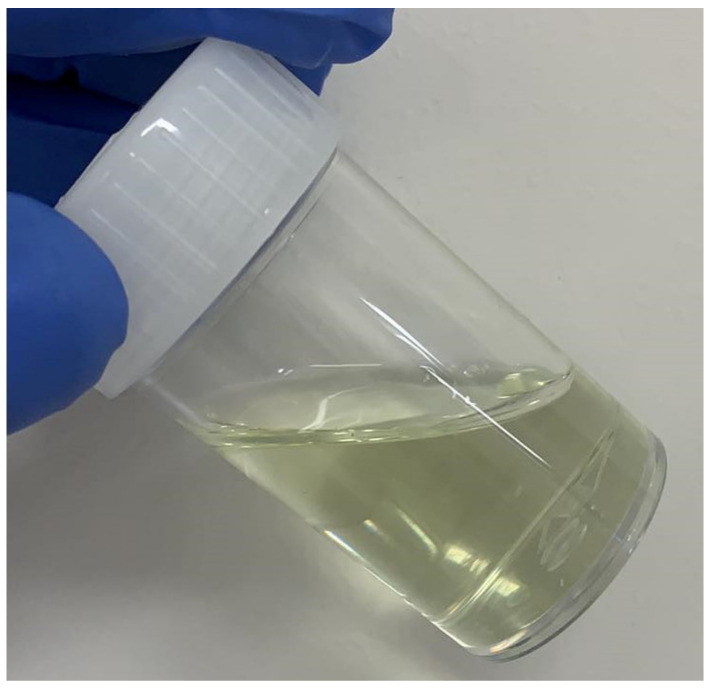
Binary PS80/P407 mixed micelles.

**Figure 3:**
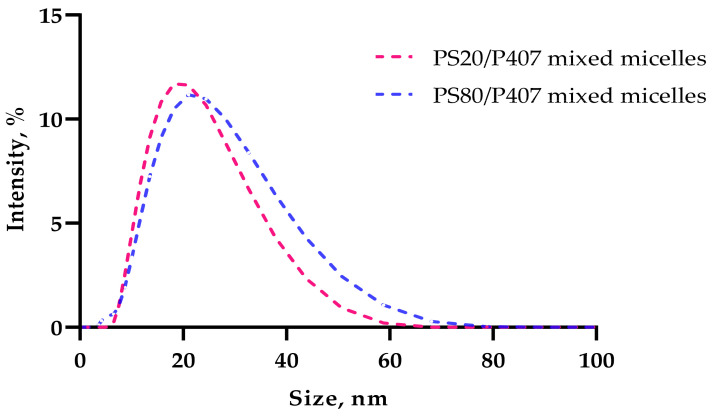
Size distribution (by intensity) of clozapine-encapsulated binary PS20/P407 and PS80/P407 mixed micelles (*n* = 3).

The saturation solubility of clozapine in 15.5% *w*/*w* P407 solution was 0.4 ± 0.04 mg/mL. Therefore, PS20 or PS80 was used to increase the concentration of clozapine to a final concentration of 1 mg/mL (0.1% *w*/*w*). As shown in Table 2, the hydrodynamic particle diameter of the binary polysorbate/poloxamer mixed micelles was shown to be <<50 nm, with PS20 producing slightly smaller particles (Figure 1) than PS80 (Figure 2). The PDI values were low for both sets of micelles and were deemed acceptable (≤0.3) [14], indicating uniformly dispersed binary mixed micelles (Figure 3). The zeta potential of the binary mixed micelles without clozapine was −0.2 ± 0.8 mV for 15.5% *w*/*w* P407 and 3% *w*/*w* PS20, and 0.3 ± 0.2 mV for 15.5% *w*/*w* P407 and 3% *w*/*w* PS80. The zeta potential of native clozapine particles (as received) was measured to be −1.0 ± 0.5 mV, and may have reduced the overall zeta potential of the drug-encapsulated binary mixed micelles. 

### 2.2. Influence of PS20 and PS80 on T_sol/gel_ and Turbidity of the Sol–Gel Platform

**Figure 4:**
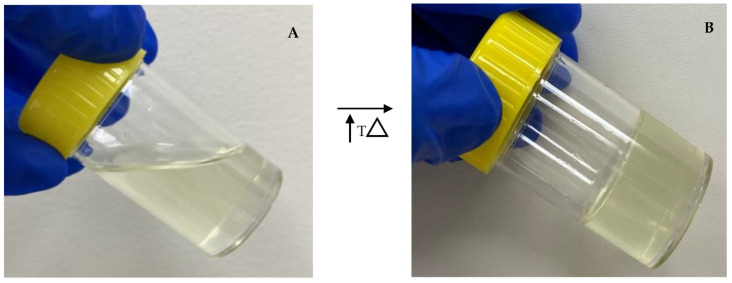
Representative photograph of F3. (**A**) *Sol* form at storage temperature 2–8 °C. (**B**) *Gel* form at nasal temperature 32−34 °C.

**Figure 5:**
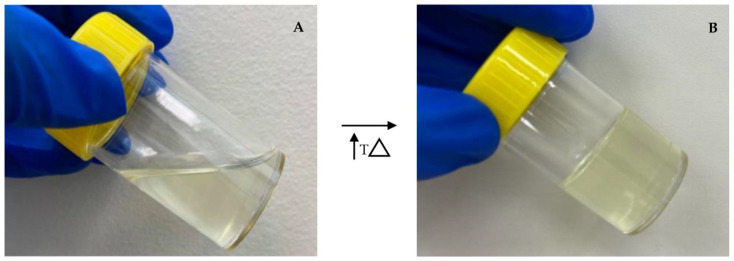
Representative photograph of F4. (**A**) *Sol* form at storage temperature 2–8 °C. (**B**) *Gel* form at nasal temperature 32−34 °C.

The addition of PS20 or PS80 in increasing concentrations led to a decreased turbidity and increased T_sol/gel_ (Table 3). Formulations with PS20 showed no significant difference (*p* = 0.41) in T_sol/gel_ to those with PS80 at the same concentration. However, the turbidity was significantly lower for formulations with PS20 (*p* = 0.04). Sol–gels with 1% *w*/*w* PS20 (F1) or 1% *w*/*w* PS80 (F2) had similar T_sol/gel_ to those without polysorbate (Control 1 and Control 2). However, the turbidity of Control 2 decreased with the addition of 1% *w*/*w* PS20 (F1) and 1% *w*/*w* PS80 (F2). The addition of 5% *w*/*w* PS20 (F5) or 5% *w*/*w* PS80 (F6) had the lowest turbidity. However, both formulations displayed a gelation above the targeted T_sol/gel_ of 28 °C. F3 and F4 had turbidities ≤25 NTU with T_sol/gels_ near 28 °C, and were therefore chosen as optimized sol–gel formulations (Figure 4 and Figure 5). 

### 2.3. Flow Behavior of Optimized Formulations

**Figure 6:**
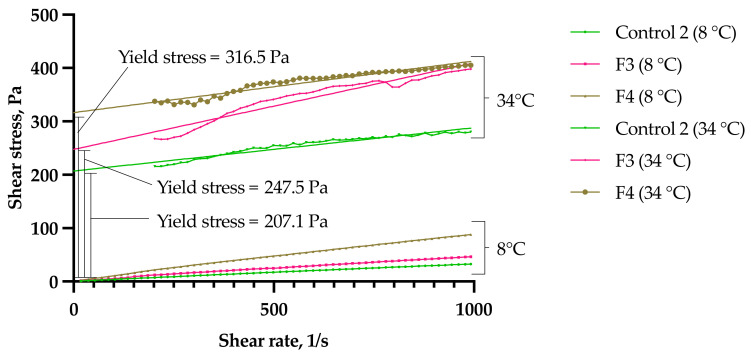
Flow rheogram of Control 2 and optimized sol–gels (F3 and F4) at storage temperature (8 °C) and nasal temperature (34 °C).

The flow characteristics of the optimized sol–gels (F3 and F4) are classified according to the relationship between shear stress and shear rate [15,16], and compared to the clozapine gel suspension without any polysorbates (Control 2) at 8 °C (storage temperature) and 34 °C (nasal temperature). According to Newton’s Law, shear stress is defined by the equation below:

(1) $$\tau =\eta \times \dot{\gamma }$$

where τ = shear stress, η = viscosity and $\dot{\gamma }$ = shear rate. The viscosity of Newtonian fluids does not change with the shear rate. The rheological studies estimate the formulations’ spreadability at lower temperatures for ease of administration, and their rigidity at higher temperatures for an increased residence time on the application site. The rheogram (Figure 6) below shows a linear shear-stress–shear-rate relationship for all three formulations at 8 °C, which suggests Newtonian flow-like behavior, indicating that they were in a solution form [17]. Control 2 showed the lowest fluctuation with an increasing shear rate, as expected without the presence of viscous PS20 or PS80. F4 showed a higher fluctuation and resistance to flow compared to F3 with an increasing shear rate, indicating that PS80 forms stronger interchain entanglements with polymer chains at low temperatures. This suggests that the addition of PS80 showed a poorer spreadability compared to PS20.

At 34 °C, the polymers exhibited solid-like gels that were resistant to deformation with shear rates below 200 s^−1^. These gels require a critical shear stress to break the structure of the gels to start flowing, with F4 having the highest yield stress of 316.5 Pa, followed by F3 at 247.5 Pa and Control 2 at 207.1 Pa. This suggests that PS80 increases the elasticity and mechanical strength of the gel more than PS20, which would be expected to enhance the retention time at the nasal mucosa [18]. As expected, Control 2 behaved more as a weakly cross-linked gel without the presence of polysorbates. Above 200 s^−1^, the polymer chains disentangle along the direction of the shear, and the polymers start to flow steadily with an increasing shear rate (Figure 6). This implies Bingham pseudoplastic behavior for all three formulations at the nasal cavity temperature. 

### 2.4. Gel Strengths, Viscosities and Loss Tangent (tan δ) of Optimized Clozapine Sol–Gel Formulations

**Figure 7:**
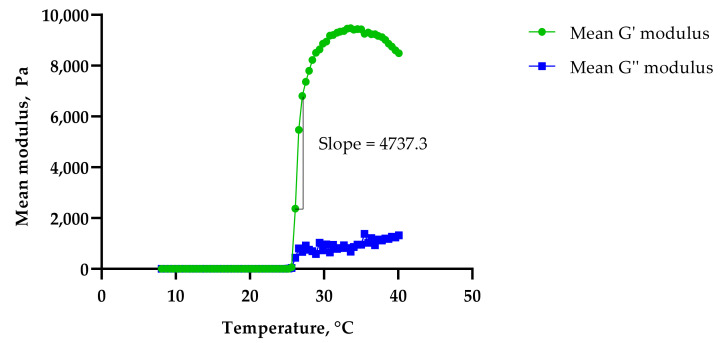
Sol–gel transition temperature for Control 2.

**Figure 8:**
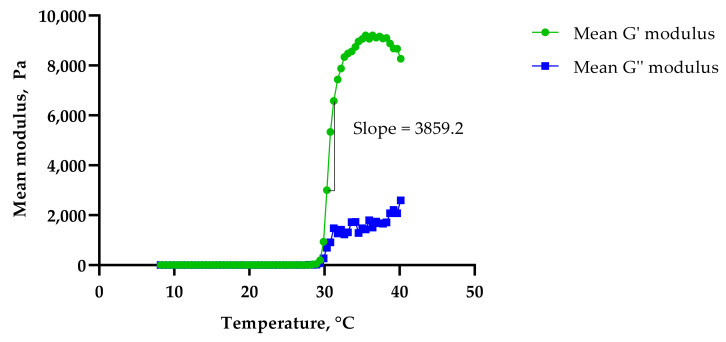
Sol–gel transition temperature for F3.

**Figure 9:**
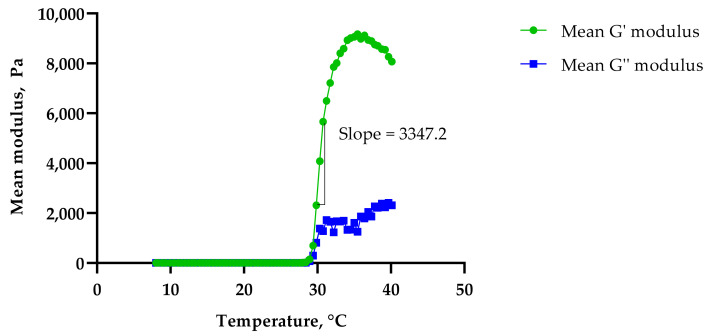
Sol–gel transition temperature for F4.

The G′ modulus (storage modulus) quantifies the energy stored and recovered after deformation per cycle at a given frequency [17], and is an indirect measure of gel strength [19,20]. The G″ modulus (loss modulus) measures the energy dissipated from the gels at a given oscillatory angle. It can be observed that both the G′ and G″ moduli and the viscosities of all three formulations were low at lower temperatures, which indicated that the formulations were in liquid or ‘*sol*’ form. As the temperature increased, the formulations became more elastic and the G’ modulus increased drastically due to the formulations’ solid gel-like mechanical spectra (G′ > G″). This is more likely due to the dehydration of the PPO block leading to the aggregation of orderly packed micelles at higher temperatures [21]. The addition of PS20 or PS80 was seen to delay the gelation response, with PS80 showing a longer drag than PS20. This was measured using the slope of the G′ modulus curve, with Control 2 showing the steepest slope of 4737.3 (Figure 7), followed by PS20 with 3859.2 (Figure 8) and PS80 with 3347.2 (Figure 9). The hydrophilic polysorbates in the formulations may have attracted more water, requiring more heat energy to dehydrate and solidify the polymer gel matrix. 

F4 showed the highest elasticity at the nasal temperature compared to F3, whereas Control 2 showed the lowest (Table 4). This may be explained by the synergism between PS20 or PS80 and P407 when micellization occurs. The degree of interaction between PS80 and P407 may be larger than PS20 and P407. The hydrophobic part of PS80 may have better enthalpic interactions with the PPO block of P407, thereby producing greater thermodynamic micelle stabilization [22]. The dynamic viscosity measures the resistance to flow at a given oscillating movement [17]. F4 showed the highest viscosity at the nasal temperature, followed by F3 and Control 2 (Table 5). This is due to the higher hydrophobicity and thicker consistency of PS80 in F4 leading to a greater resistance to flow in a gel-structured state, compared to PS20 for F3 and no polysorbates for Control 2. The value of the phase angle (tan δ = G″/G′) is used to understand the internal structure of the formulations, which describes the ratio between the viscous components and the elastic components [23]. The tan δ was more than 1 (G″ > G′) at lower temperatures for all formulations, demonstrating liquid-like (viscous) properties. As the temperature increased, the tan δ became less than 1 (G″ < G′), showing solid-like (elastic) properties (Table 6). Therefore, the smaller the tan δ, the higher the elasticity of the formulation [17]. F4 showed more elastic properties than F3 and Control 2 (Table 4 and Table 6). 

### 2.5. DSC and TGA of Optimized Formulations

**Figure 10:**
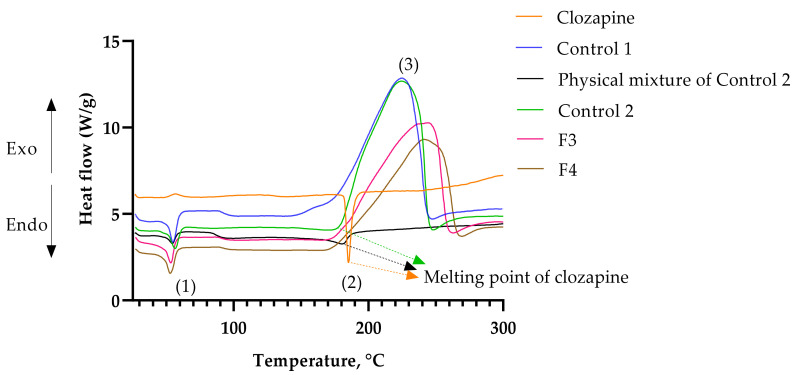
DSC thermogram. (1) indicates the melting peaks of P407, (2) melting peaks of clozapine, and (3) oxidative degradation peaks of the formulations.

**Figure 11:**
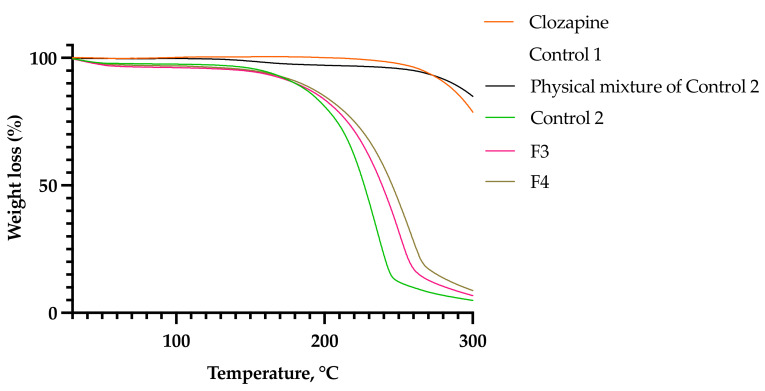
TGA thermogram.

DSC and TGA analyses were performed in order to evaluate the thermal kinetics of the optimized formulations. The physical mixture of Control 2 was evaluated in comparison to the formulated Control 2, whereby clozapine 0.1% *w*/*w* was added to the sol–gel platform, stirred using a spatula for 10 s and immediately analyzed. The first endothermic peak (1) in the DSC thermogram (Figure 10) shows the melting point of P407. As expected, the addition of clozapine powder increased the melting point of the polymer sol–gel platform from 54.9 °C (Control 1) to 56.6 °C (Control 2), but not for the physical mixture of Control 2 (54.2 °C). On the other hand, the addition of clozapine dissolved in PS20 and PS80 liquids decreased the melting point of the sol–gel to 53.2 °C (F3) and 52.7 °C (F4), respectively. The endothermic peak (2) of the raw clozapine curve represents the melting point of the drug at 185.2 °C. The physical mixture of Control 2 reduced the melting point of clozapine and showed a broad endothermic peak at 180.1 °C, whereas Control 2 showed a broader peak at 169.9 °C. F3 and F4 did not show any melting peaks, indicating drug encapsulation or amorphous dispersions of clozapine in the sol–gels. The exothermic peaks at (3) in the DSC curve (ranged between 147–245 °C for Control 1, 179–248 °C for Control 2, 171–266 °C for F3 and 169–270 °C for F4) represent auto-oxidation, which corresponds with the weight loss in the TGA curve. This may be due to the eruption of POE chains, consequently producing oxidative byproducts and volatile degradants [24]. The absence of an exothermic peak at (3) for the physical mixture of Control 2 indicates a lack of interaction between the polymer sol–gel platform and clozapine. The addition of PS80 produced shorter POE chains upon cleavage compared to the addition of PS20, as evident through the fronting of the exothermic peak (3) [25]. The optimized formulations, F3 and F4, showed that the addition of PS20 or PS80 produced weaker exothermic reactions (Figure 10). In the TGA thermogram, the onset of weight loss was also delayed, with F4 occurring at a higher temperature than F3 (Figure 11). This implies that more heat energy is required to set about oxidation with the addition of PS80 as compared to PS20. Although unsaturated fatty acids are more susceptible to oxidation compared to their saturated counterparts [26], the higher heat energy required to rupture the polymer chains could be explained by the higher thermodynamic micelle stabilization of PS80 and P407. From the data, it can be concluded that the auto-oxidation is primarily caused by the cleavage of the POE chains, and the longer hydrocarbon structure (C17) of the oleate moiety in PS80 prolonged degradation compared to the shorter hydrocarbon chain (C11) of the laureate moiety in PS20.

**Figure 12:**
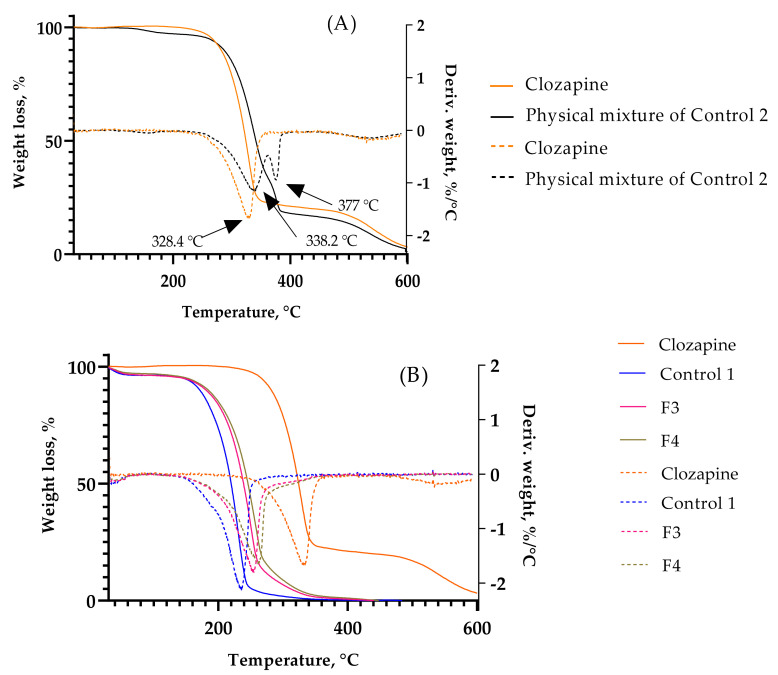
TGA (solid line) and DTG (dashed line) of (**A**) clozapine and physical mixture of Control 2, and (**B**) clozapine, Control 1, F3 and F4.

Figure 12 shows the first derivative thermogravimetry (DTG) of the TGA plots. From Figure 12A, it can be observed that the major degradation peak of clozapine occurred at 328.4 °C at a rate of −1.7%/°C. The physical mixture of clozapine and the polymer matrix (Control 2) showed two decomposition processes in the DTG plot at rates slower than clozapine alone (−1.1%/°C at 338.2 °C and −0.9%/°C at 377 °C). In Figure 12B, the decomposition of all excipients took place in one step, and the absence of the DTG curve of clozapine in F3 and F4 confirms the amorphic nature of the drug in the formulations. The polymer matrix without clozapine (Control 1) degraded at a rate of −2.1%/°C at 229.5 °C. The addition of PS80 showed a slower degradation rate of −1.6%/°C at 258.1 °C compared to PS20 (−1.8%/°C at 248.3 °C). The decomposition of the formulations at such high temperatures indicates a high thermal stability, with F4 being sturdier than F3.

### 2.6. XRD Analysis

**Figure 13:**
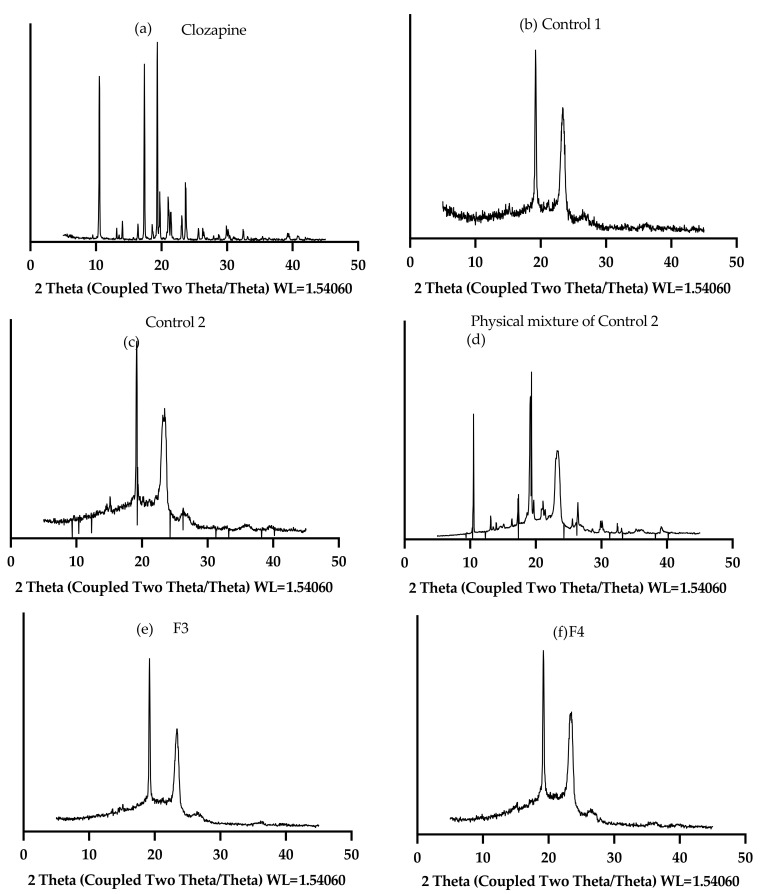
XRD patterns of clozapine, Control 1, Control 2, physical mixture of Control 2, F3 and F4.

The XRD patterns of clozapine, Control 1, Control 2, physical mixture of Control 2, F3 and F4 are displayed in Figure 13. The crystalline clozapine showed strong peaks at diffraction angle 2θ (10.5°, 17.4° and 19.4°). Control 1 (without clozapine) showed two peaks at 19.3° and 23.4°, which represent characteristic peaks of P407 [27]. The crystalline peaks of clozapine at 10.5° and 19.3° were detectable in the physical mixture of Control 2, although the intensities were decreased due to the dilution effect of the formulation. The peak of clozapine at 10.5° was not evident on the diffractogram of Control 2 but the increased intensity of the peak at 19.3° may indicate a combination of both clozapine and P407 after stirring overnight. Both F3 and F4 did not show any crystalline peaks of clozapine, demonstrating that clozapine was amorphic in PS20 and PS80, respectively.

### 2.7. In Vitro Drug Release Study

**Figure 14:**
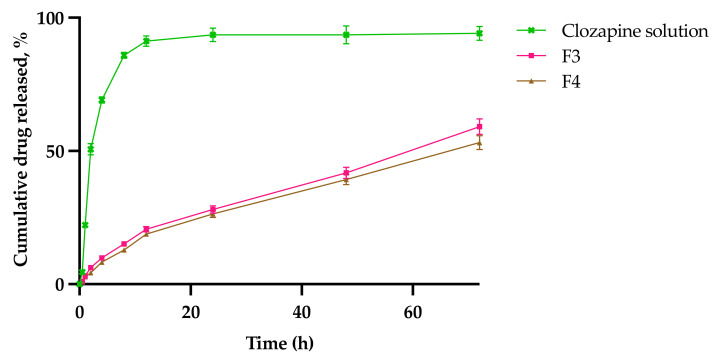
Cumulative percentage of clozapine released from clozapine solution, F3 and F4 gel matrix at 34 °C.

Figure 14 shows the release profiles of clozapine from the optimized sol–gels (F3 and F4) and compared to the clozapine solution at 34 °C. The cumulative percentage release of clozapine was 86.8 ± 1.1% at 8 h for the clozapine solution. Contrarily, the sol–gels showed a slow, extended cumulative release of clozapine, with F4 (53.1 ± 2.7%) being slower than F3 (59.1 ± 3.0%) at 72 h (*p* = 0.01). The release rates of clozapine from F3 (76.3 ± 3.8 μg/cm^2^ h^−1^, *p* < 0.05) and F4 (69.8 ± 3.5 μg/cm^2^ h^−1^, *p* < 0.05) were also shown to be significantly slower than that of the clozapine solution (337.9 ± 7.2 μg/cm^2^ h^−1^). These findings demonstrate that PS80 increased the rigidity of the formulation, leading to a lower amount of the drug and a slower rate of drug release from the polymer gel matrix at 34 °C. The increased residence time between clozapine and the nasal mucosa is desirable to prevent drug degradation via mucociliary clearance and the rapid turnover of mucus in the nasal cavity [28].

The R^2^ was highest in the Korsmeyer–Peppas model for both F3 and F4, and was therefore considered to be the best model (Table 7). All experimental release data for F3 and F4 were plotted and fitted into Equation (8), since both curves fall within the 60% cut-off value at 72 h [29,30]. The *n* was calculated to be 0.5 < *n* < 1 for both F3 and F4. This indicates that the in vitro dissolution study showed a non-Fickian, anomalous behavior release of clozapine from the sol–gel at 34 °C [31], which involved a combination of mechanisms, including the swelling of the polymer matrix and/or relaxation of polymeric chains, and drug diffusion or solvent transport across the sol–gel [30].

### 2.8. Ex Vivo Drug Permeation Study

**Figure 15:**
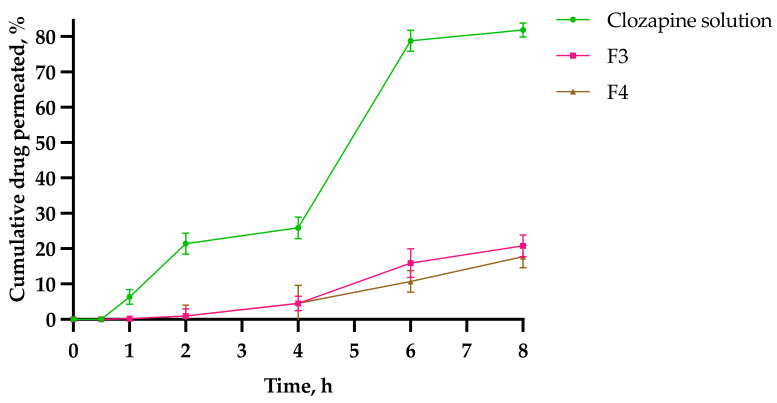
Cumulative percentage of clozapine permeation across nasal mucosal tissues from clozapine solution, F3 and F4 gel matrix at 34 °C (*n* = 3 ± SD).

Sheep nasal mucosa represents similar histological and morphological structures to human nasal mucosa and can thus be used for permeation studies [31,32,33,34]. Ex vivo permeation studies were conducted across excised sheep nasal tissues mounted onto vertical Franz diffusion cells to determine the transmucosal delivery of F3 and F4, and compared to a simple solution of clozapine. Figure 15 shows the cumulative drug permeated through the mucosa as a function of time. There was no permeation observed in the first 0.5 h for the clozapine solution, F3 and F4. The results revealed a burst drug release at 4–6 h for the clozapine solution (78.8 ± 3.0%), whereas F3 and F4 showed a sustained delivery of clozapine up to 8 h. At 8 h, 81.9 ± 2.0% of the clozapine solution was permeated through, whereas only 20.8 ± 3.0% and 17.8 ± 3.1% of clozapine permeated from F3 and F4, respectively. This demonstrated that PS80 prolonged the permeation process, assumedly due to the higher gel rigidity of F4 at 34 °C.

The permeability parameters for the 0.1% clozapine solution (control), F3 and F4 are listed in Table 8. The steady state flux for the clozapine solution was approximately 3–4 times higher than F3 and F4, which can be expected without the presence of the polymer gel matrix at 34 °C. The apparent permeability of the clozapine solution was high, which indicates its suitability for intramucosal nasal delivery. The addition of PS20 was significantly higher than PS80 for the steady state flux (*p* = 0.02), apparent permeability (*p* = 0.02), diffusion coefficient (*p* = 0.02) and cumulative clozapine permeated at 8 h (*p* = 0.002). Based on the data in Section 2.7 and Section 2.8, it is evident that the rate-limiting step for both formulations is in the permeation process.

### 2.9. Nasal Mucosal Tissue Deposition of Optimized Sol–Gels

**Figure 16:**
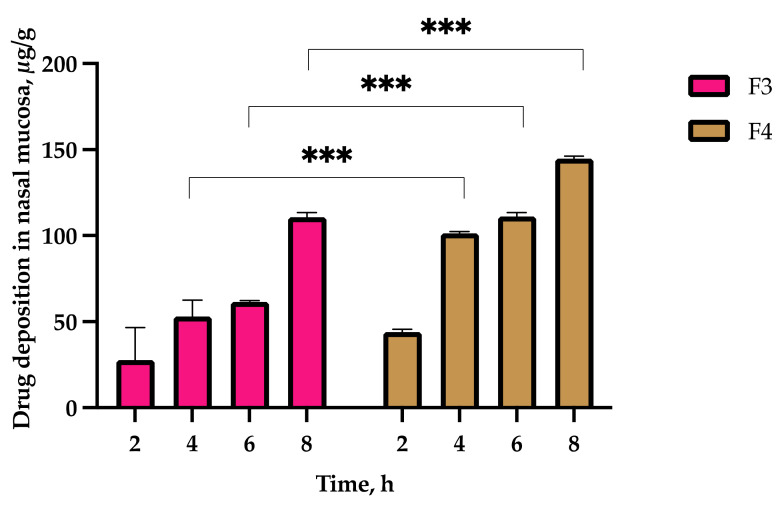
Clozapine deposition in sheep nasal mucosa from F3 and F4 up to 8 h (*n* = 3 ± SD). *** *p* ≤ 0.001.

Figure 16 shows the deposition of clozapine from F3 and F4 up to 8 h. The retention of clozapine in the nasal mucosa was significantly higher with F4 compared to F3 at 4 h (101.35 ± 1.04 µg/g vs. 53.05 ± 9.60 µg/g, *p* = 0.001), 6 h (111.21 ± 2.16 µg/g vs. 61.51 ± 0.69 µg/g, *p* < 0.001) and 8 h (144.82 ± 1.41 µg/g vs. 110.74 ± 2.74 µg/g, *p* < 0.001), but not at 2 h (44.07 ± 1.54 µg/g vs. 27.68 ± 18.97 µg/g, *p* = 0.21). This could be due to a lag phase, where no drug was permeated in the first 0.5 h and the controlled diffusion of clozapine from the polymer gel matrix occurred through the tissues 2 h post-application. The lower permeation of F4 may be attributed to its higher drug retention in the mucosal tissues.

### 2.10. Drug Stability Study

As shown in Table 9, the stability study data indicated that the drug content decreased to approximately 86–88% at 8 °C, 76% at 25 °C and 69–73% at 40 °C over 3 months. At higher temperatures, the desolvation, dehydration and evaporation of water in P407 solutions take place, and POE crystals may precipitate in the gel during dehydration, affecting the solubility of clozapine in the formulation over time [35]. The zeta potential values of the binary mixed micelles were low at −2.7 ± 1.1 mV for PS20-P407 and −3.0 ± 0.8 mV for PS80-P407. The stability of the micelles could be attributed to the hydrophilic groups of P407 and polysorbates, which may have provided a protective barrier and promoted steric stabilization by retarding Ostwald ripening and coalescence [36,37]. Upon visual inspection and on centrifugation at 12,000 rpm for 30 min at 4 °C, the sol–gels exhibited no phase separation after each time point.

Clozapine has a low water solubility of 11.8 µg/mL at 25 °C [38,39,40]. This study demonstrated that 15.5% P407 alone increased its solubility to 0.36 ± 0.04 mg/mL. The optimized sol–gels, which were also incorporated with 3% PS20 or 3% PS80, increased the solubility of clozapine by over 80-fold. Moreover, clozapine has a pKa of 7.5 [38,39,40], which makes it more polar and ionizable in acidic environments, such as that of the nasal cavity. The binary polysorbate/poloxamer mixed micelles in this study produced the desired particle size for N2B delivery, as they were considerably smaller than the 100–700 nm axon diameter (in humans) [41]. This may potentially alleviate peripheral ADRs by minimizing drug exposure in peripheral tissues without compromising the drug’s therapeutic effect.

**Figure 17:**
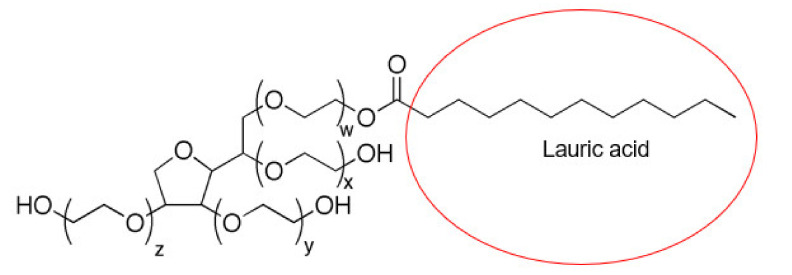
Chemical structure of PS20.

**Figure 18:**
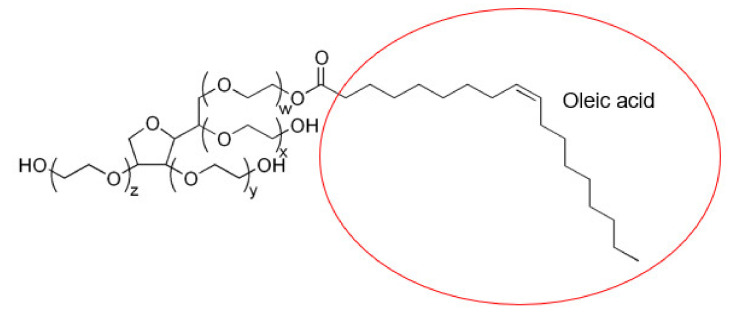
Chemical structure of PS80.

P407 (EO_100_PO_65_EO_100_) contains a PPO/PEO composition ratio of 0.34 and has a critical micelle concentration (CMC) of 0.7% *w*/*v* at 25 °C, 0.1% *w*/*v* at 30 °C and 0.025% *w*/*v* at 35 °C (11). P407 sol–gels are negative thermoresponsive polymers [42], making it a good solvent below the lower critical solution temperature (LCST). Above the LCST, phase separation occurs and P407 polymers become hydrophobic and insoluble in aqueous solutions [42]. The phase behavior of P407 is dependent on its concentration and temperature. Above the CMC and critical micellization temperature (CMT), the micellization of P407 is driven by the hydrophobic PPO block due to entropy [11]. The synergism of the binary polysorbate/poloxamer surfactant mixture decreased the overall CMC due to enthalpy, and the enthalpic interactions between the two surfactants produced a system that is more thermodynamically stable, generating stronger micellar sol–gels. The two polysorbates used in this study have the same polar group but different hydrophobic segments. Therefore, the differences seen between F3 (Figure 17) and F4 (Figure 18) can be attributed to the alkyl moieties and their arrangement within the core of the P407 micelles [22].

Oleic acid in PS80 has more carbon atoms, thus producing more hydrophobic interactions with the PPO core of P407. However, the double bond at C9 was reported to form weaker interactions with micelles due to steric hindrance [22]. On the other hand, the saturated moiety of lauric acid may have improved micellar packing, although the weaker synergism observed with F3 compared to F4 could be offset by the lower number of hydrocarbon chains of lauric acid. This is proven by the small difference in T_sol/gel_ between F3 and F4. Moreover, the hydrophilic–lipophilic balance (HLB) of PS20 is higher than PS80, which makes PS80 more favorable to the PPO units of P407.

## 3. Conclusions

The addition of PS20 or PS80 markedly improved the drug loading, micellar packing, increased stabilization and mechanical strength of the P407-based sol–gel formulations. Formulation F4 was found to be more elastic than F3 at the nasal temperature due to the higher synergism of binary PS80-P407 mixed micelles, producing more thermodynamically stable and rigid micellar sol–gels than PS20-P407. The variations in encapsulation, drug release and drug permeation recorded between F3 and F4 did not consider the particle size, PDI and zeta potential, as they were not significantly different. Using the Korsmeyer–Peppas model, our results showed a non-Fickian, anomalous behavior release of clozapine from the sol–gel at 34 °C, which involved a combination of mechanisms, including polymer swelling and drug diffusion. The permeation of clozapine in F3 through the nasal mucosa tissue was shown to be better, with more of the drug retained within the tissue post-administration of F4. Therefore, binary PS20-P407 mixed micelles in a sol–gel system may be a preferable carrier system for intranasal clozapine delivery to the brain compared to binary PS80-P407 mixed micelles.

## 4. Materials and Methods

Clozapine (>98% purity) was purchased from Adooq Bioscience (Irvine, CA, USA). P407, hydroxypropyl methyl cellulose (HPMC E4M) and glycerin were purchased from Sigma-Aldrich (Castle Hill, NSW, Australia). PS20, PS80 and carbopol 934 NF were purchased from PCCA (Matraville, NSW, Australia). Hydrochloric acid (HCl), sodium hydroxide (NaOH), potassium chloride (KCl), calcium chloride dihydrate (CaCl_2_·2H_2_O), sodium chloride (NaCl) and methanol were of analytical grade and purchased from Sigma-Aldrich (Castle Hill, NSW, Australia). Orthophosphoric acid was purchased from Thermo Fisher Scientific (Brisbane, QLD, Australia) and potassium dihydrogen orthophosphate anhydrous was purchased from Chem-Supply (Gillman, SA, Australia). Milli-Q water was used as a formulation vehicle.

### 4.1. Sol–Gel Preparation

The sol–gels were prepared using the cold method [43] as described by Pandey et al. [44] with slight modifications. Briefly, 15.5% *w*/*w* P407 and 0.5% *w*/*w* HPMC were dry mixed before sufficient volume of Milli-Q water was added to hydrate the mixture. Separately, a stock solution of 1% *v*/*v* carbopol in Milli-Q water was prepared. Then, 3% *w*/*w* glycerin and 0.1% *w*/*w* of the carbopol solution were added to the mixture, and stirred thoroughly (400 rpm) for 6 h at 2–8 °C. At the same time, 0.1% *w*/*w* clozapine was stirred in 1%, 3% or 5% *w*/*w* PS20 or PS80 at room temperature before being mixed with the polymer solution, and the final weight of the formulation was made up to 10 g with Milli-Q water and stirred thoroughly (400 rpm) overnight at 2–8 °C. The final sol–gel mixture was adjusted to pH 5.5 ± 0.2 with 0.1 M HCl or 0.1 N NaOH.

### 4.2. Determination of Clozapine Saturation Solubility in ‘Sol’ form of 15.5% w/w P407

An excess amount of clozapine was added to 15.5% *w*/*w* P407 and stirred overnight (400 rpm) at 2–8 °C. Samples were centrifuged at 12,000 rpm at 4 °C for 45 min using a refrigerated centrifuge (Eppendorf Centrifuge 5804 R, Hamburg, Germany). The supernatant liquid was collected and filtered using a 0.45 µm, 25 mm PTFE Syringe Membrane Filter (PhaseSep Pty Ltd., Doncaster East, VIC, Australia), and the concentration of clozapine was measured using HPLC [45,46].

### 4.3. Preparation of Simulated Nasal Fluid (SNF)

SNF was prepared by dissolving KCl 1.29 mg/mL, NaCl 7.45 mg/mL and CaCl_2_·2H_2_O 0.32 mg/mL with Milli-Q water, and was adjusted to pH 5.5 ± 0.1 with 0.1 M HCl and 0.1 N NaOH [47,48].

### 4.4. Rheology Studies

Rheological evaluations of all sol–gel formulations were performed using a Discovery Hybrid Rheometer HR-3 (TA Instruments, New Castle, DE, USA) with a 40 mm parallel plate geometry and a sample gap of 200 μm. The gelation temperature (T_sol/gel_), G’ modulus, G’’ modulus and loss tangent (tan δ) of the sol–gels were evaluated using oscillatory measurements, with a temperature ramp between 8 °C to 40 °C and ramp rate of 5 °C/min at 1.0 Pa (stress) and 1.0 Hz (frequency). The dynamic viscosity of the sol–gels was measured using flow measurements, with a temperature ramp between 8 °C to 40 °C and ramp rate of 5 °C/min at 2.0 rad/s (angular velocity). The flow behavior of the sol–gels was evaluated at 4 °C (storage temperature) and 34 °C (nasal temperature), with a flow ramp between 10 s^−1^ to 1000 s^−1^. The strength of the gels at nasal temperature was recorded using the G’ modulus as a measurement of stiffness. All measurements were carried out in triplicate and the resultant curves were generated directly from the manufacturer’s computer TRIOS software (TA Instruments, New Castle, DE, USA).

### 4.5. Determination of Turbidity

The turbidity of the sol–gels when in liquid form was performed using a Hach TU5200 EPA Turbidimeter (Hach Company, Loveland, COL, USA) measuring in nephelometric turbidity units (NTU) with a Class 2 laser product and 650 nm (EPA 0.43 mW) optical light source. The sol–gels were left at room temperature and any air bubbles were removed before evaluation. The scattered light was collected at a 90° angle to the incident light and 360° around the sample vial. The turbidity sensor was calibrated using three Stablcal^®^ turbidity standards (10, 20, 600 NTU). The relative clarity of the sol–gels was measured in triplicate.

### 4.6. Particle Size, PDI and Zeta Potential

The hydrodynamic particle diameter, PDI, and zeta potential of clozapine in the binary polysorbate/poloxamer mixed micelles were measured using dynamic light scattering (DLS) with Zetasizer Nano ZS (Malvern Instruments, Malvern, UK). Diluted samples (1:10) were pipetted into disposable cuvettes for particle size and PDI measurements, and disposable DTS1070 folded zeta cells (Malvern Instruments, Malvern, UK) for zeta potential measurements. The detection scattering angle was set at 173° (25 °C) with equilibration time at 60 s. Measurements were carried out in triplicate and recorded as the mean ± SD of three independent runs.

### 4.7. High Performance Liquid Chromatography (HPLC) Analysis and Quantitation of Clozapine

The concentration of clozapine was analyzed using a RP-HPLC equipment (Shimadzu Nexera-i LC-2040C, Kyoto, Japan) equipped with a low pressure quaternary gradient pump, along with a dual wavelength UV detector (at 234 nm), auto sampler (10 μL injection volume) and column oven (maintained at 25 °C). The chromatographic data were processed using LC solution 1.24 SP1 software. The quantification analysis was performed under isocratic conditions with a C18 column (Phenomenex Gemini C18 150 × 2 mm, 3 μm). The mobile phase consisted of methanol and phosphate buffer (3.4 mM potassium dihydrogen orthophosphate buffer, pH 2.0 adjusted with 10% *v*/*v* o-phosphoric acid) in the ratio of 65:35 (*v*/*v*). The flow rate was 0.2 mL/min and the retention time of clozapine was found to be 3.3 min. The concentration of clozapine was calculated using a standard calibration curve (R^2^: 0.9998) over the concentration range of 1–50 μg/mL. The lower limit of detection (LOD) was 0.03 μg/mL and the lower limit of quantification (LOQ) was 0.1 μg/mL.

### 4.8. Differential Scanning Calorimetry (DSC) and Thermogravimetric Analysis (TGA)

The sol–gels were frozen with liquid nitrogen for an hour and then lyophilized using a VirTis BenchTop Pro with Omnitronics™ freeze dryer (SP Industries, Warminster, PA, USA) at −102 °C (50 Hz) and ≤20 mT for 24 h. The powdered sol–gels of approximately 4–5 mg were transferred into aluminum crucibles and the thermal analysis of the sol–gels was carried out using a Mettler Toledo TGA/DSC 2 equipment (Mettler Toledo, Columbus, OH, USA). Heating runs were performed under compressed air, with a heating rate of 10 °C/min over 25–600 °C (49). STARe software was used to generate the thermal curves by measuring heat output (DSC) and mass loss (TGA) as temperature is increased.

### 4.9. X-ray Power Diffraction (XRD) Analysis

X-ray diffraction was carried out to identify phases in the powdered sample, with data collected using a Bruker D8 Advance MKII XRD X-ray diffractometer (Bruker, Billerica, MA, USA) equipped with a Cu source, a LynxEye detector and operated at 40 kV and 40 mA. Diffraction patterns were recorded by continuous scans from 5 to 45° 2Ɵ, with a step size of 0.04° and 15 rpm rotation at a scan rate of 0.4° s per step. The resulting patterns were imported into Diffrac EVA version 5.1, where phases were identified using the PDF-4 2020 ICDD database [49].

### 4.10. In Vitro Drug Release Study

In vitro drug release studies were performed using a Logan DHC-6T vertical Franz diffusion apparatus (Logan instruments, Somerset, NJ, USA). Clozapine solution (0.1% *w*/*w*) was used as control, prepared in 1 mL 0.1 M HCl and made up to 10 g with Milli-Q water (adjusted to pH 5.5 with 0.1 N NaOH). The donor compartment was filled with 1.5 mL of the optimized sol–gel or clozapine solution, and the receiver compartment was filled with 12 mL of SNF. Snake skin dialysis membranes (Thermo Fisher Scientific, Brisbane, QLD, Australia) with a 3.5 kDa MWCO were soaked in SNF for 15 min prior to experimentation. Membranes having effective areas of 1.5 × 1.5 cm^2^ exposed to the test formulations/solutions were then mounted between the donor cap and receiver body. The temperature of the chamber was kept at 34 ± 1 °C with standard stirring speed. Samples of 0.5 mL were collected from the receiver compartment at 0, 0.5, 1, 2, 4, 8, 12, 24, 48 and 72 h, and replaced with fresh SNF of equal volume after each sampling. The samples were placed in a −80 °C refrigerator until analysis using HPLC. The release study experiments were performed in triplicate.

### 4.11. Ex Vivo Drug Permeation Study

Ex vivo drug permeation of the optimized sol–gels was carried out using Franz diffusion apparatus using excised sheep nasal mucosal tissues [31,50], which were obtained from The University of Queensland (UQ)’s School of Veterinary Science (Gatton, QLD, Australia) following a process supervised by veterinary officials in accordance with the Ethics Committee of Animal Experimentation at UQ (Ethics Approval No. 2021/AE000143). The mucosal specimens, with effective surface area of 1.5 × 1.5 cm^2^, were mounted onto the Franz diffusion apparatus, with the mucosal surface facing the donor compartment and serosal side facing the receptor compartment. The donor compartment was filled with 500 μL of the optimized sol–gels or clozapine solution (as described above), and the receiver compartment was filled with 12 mL of SNF to ensure sink conditions. The temperature of the chamber was kept at 34 ± 1 °C with standard stirring speed. The tissues were allowed to stabilize for 30 min prior to loading of the optimized sol–gels. Sample aliquots of 0.5 mL were collected from the receiver compartment at 0, 1, 2, 4, 6, 8 h and replaced with fresh SNF of equal volume after each sampling. The samples were placed in a −80 °C refrigerator until analysis using HPLC. The experiments were carried out in triplicate and the data were fitted into Fick’s second law of diffusion equation to determine the permeability parameters of clozapine in the sol–gels across the sheep nasal mucosa, where C*_t_* is the cumulative drug permeated at time *t*, *C_d_* is concentration of clozapine in the donor chamber (0.5 mg), *K* is the partition coefficient (log P) of clozapine, *L* is the diffusion path length and *D* is the diffusion constant, which was calculated using Equation (3) [31,51].

(2) $$C_{t}=C_{d}\cdot \left(KL\right)\{\begin{array}{c} \frac{D}{L^{2}} \end{array}\cdot t-\frac{1}{6}-\frac{2}{\pi^{2}}\;\sum_{n=1}^{\infty }\frac{{\left(-1\right)}^{n}}{n^{2}}\cdot exp(-\frac{D}{L^{2}}\cdot n^{2}\cdot \pi^{2}\cdot t)\}$$

*D* = *P_app_ · L/K* (3)

The apparent permeability coefficient, *P_app_*, was calculated using Equation (4), and the steady state flux, *Jss*, was calculated using Equation (5), where *S* represents the cross-sectional area of flow.

*P_app_* = *J_ss_/C_d_* (4)

*J_ss_* = Δ*C_t_*/Δ*t*·*S* (5)

### 4.12. Mathematical Modelling of In Vitro and Ex Vivo Drug Release Kinetics

The in vitro and ex vivo drug release data were fitted to mathematical models, including zero-order, first-order, Hixson–Crowell and Korsmeyer–Peppas, to predict the kinetics and release mechanism of clozapine from the sol–gel polymer matrix [30]. For zero-order kinetics, the release of clozapine can be described using Equation (6), where *C*_0_ is the initial concentration of clozapine released (usually, *C*_0_ = 0) and *K*_0_ is the zero-order constant.

*C_t_* = *C*_0_ + *K*_0_*t* (6)

For first-order kinetics, Equation (7) was used to describe the release of clozapine from the polymer gel matrix, where *K*_1_ is the first-order constant.

*Log C_t_* = *Log C*_0_ + (*K*_1_*t*/2.303) (7)

Equation (8) illustrates the release of clozapine using the Hixson–Crowell model, where *K_HC_* is the Hixson–Crowell constant.

∛*C_t_* = ∛*C*_0_ + *K_HC_t* (8)

For Korsmeyer–Peppas kinetics, drug release can be described using Equation (9), where *F* represents the fraction of drug released at time *t*, *M_t_* is the amount of drug released at time *t*, *M_∞_* is the amount of drug released at infinity, *K_m_* is the Korsmeyer–Peppas constant and *n* is the diffusion or release component [31]. In this model, if *n* = 0.5 (Fickian Case I), the drug release is driven by diffusion, and when *n* = 1 (non-Fickian Case II), the drug release is governed by polymer swelling or relaxation of polymeric chains. When 0.5 < *n* < 1 (non-Fickian anomalous), the drug release process is indicated by both diffusion and swelling of the polymer matrix. If *n* > 1 (non-Fickian Super Case II), the drug release is said to be due to the tension and breaking of the polymer [30].

*F* = (*M_t_/M_∞_*) = *K_m_·t^n^* (9)

The obtained regression (R^2^) values were used to verify the release of clozapine from the sol–gel formulations, and the highest correlation coefficient was considered to be the best model.

### 4.13. Nasal Mucosal Tissue Deposition of Optimized Sol–Gels

The mucosal tissues were then collected at 2, 4, 6 and 8 h following exposure of the optimized sol–gels or control solution. The collected tissues were washed with Milli-Q water, blotted with filter paper, wrapped in aluminum foil and stored at −80 °C until analysis. On the day of analysis, the tissues were thawed and dried with filter paper. They were then frozen with liquid nitrogen and grinded using a mortar and pestle. Acetonitrile was added to the powdered tissues to make 100 mg/mL of tissue homogenate. The samples were vortexed for 10 s and centrifuged at 10,000 rpm for 5 min. The supernatant was collected for HPLC analysis to determine the concentration of clozapine deposited in the nasal tissues [44].

### 4.14. Drug Stability Study

The optimized sol–gel formulations were subjected to drug stability study for a period of three months at low temperature (2–8 °C), room temperature (25 °C) and elevated temperature (40 °C). Phase separation was determined after refrigerated centrifugation (12,000 rpm for 30 min at 4 °C) at one, two and three months for each temperature to observe for time-dependent changes in drug content [30,52].

### 4.15. Data Analysis

The data analysis was measured using *t*-test to determine statistical differences between individual means. In all analyses, a two-tailed *p*-value < 0.05 denotes significance. All analyses were performed using GraphPad Prism v9.

## Figures and Tables

**Table 1:** Increasing T_sol/gel_ with decreasing concentrations of P407.

Concentration of P407, % *w*/*w*	T_sol/gel_, °C
16.5	24.5 ± 0.7
15.5	28.6 ± 0.2
14.5	30.8 ± 1.3
13.5	37.0 ± 0.9

**Table 2:** Hydrodynamic particle diameter, PDI and zeta potential of clozapine in binary mixed micelles.

Binary Mixed Micelles	Hydrodynamic Particle Diameter, nm	PDI	Zeta Potential, mV	Count Rate, kcps
15.5% *w*/*w* P407 and 3% *w*/*w* PS20	18.7 ± 0.2	0.3 ± 0.02	−2.7 ± 1.1	287.1
15.5% *w*/*w* P407 and 3% *w*/*w* PS80	20.0 ± 0.4	0.3 ± 0.004	−3.0 ± 0.8	337.5

**Table 3:** Formulations containing a range of PS20 and PS80, and their respective T_sol/gel_ and NTU values.

Formulation ^#^	Solubilizers	T_sol/gel_, °C	Turbidity, NTU
Control 1 (without clozapine)	-	25.8 ± 0.5	18.4 ± 0.4
Control 2 (with clozapine)	-	25.7 ± 0.4	114.3 ± 0.6
F1	1% PS20	25.7 ± 0.6	63.4 ± 0.3
F2	1% PS80	24.8 ± 0.2	67.0 ± 0.9
F3	3% PS20	29.0 ± 0.8	22.0 ± 0.1
F4	3% PS80	28.5 ± 0.2	25.0 ± 0.1
F5	5% PS20	32.4 ± 1.1	18.4 ± 0.4
F6	5% PS80	32.7 ± 0.9	24.2 ± 0.1

^#^ All formulations contain 15.5% *w*/*w* P407, 0.5% *w*/*w* HPMC, 3% *w*/*w* glycerin, 0.1% *w*/*w* carbopol and adjusted to pH 5.5 ± 0.2. All formulations, except Control 1, contain 0.1% *w*/*w* clozapine.

**Table 4:** Mean G’ modulus of Control 2, F3 and F4 at different temperatures.

Formulation	Mean G’ Modulus at 8 °C, Pa	Mean G’ Modulus at 25 °C, Pa	Mean G’ Modulus at 32 °C, Pa	Mean G’ Modulus at 34 °C, Pa
Control 2	0.4 ± 0.04	178.6 ± 3.3	8362.8 ± 468.4	8566.3 ± 454.0
F3	0.57 ± 0.1	0.4 ± 0.01	7881.3 ± 501.1	8746.3 ± 494.5
F4	0.3 ± 0.02	0.4 ± 0.04	8015.4 ± 454.0	9014.6 ± 491.4

**Table 5:** Mean viscosities of Control 2, F3 and F4 at different temperatures.

Formulation	Mean Viscosity at 8 °C, Pa·s	Mean Viscosity at 25 °C, Pa·s	Mean Viscosity at 32 °C, Pa·s	Mean Viscosity at 34 °C, Pa·s
Control 2	0.03 ± 0.01	0.4 ± 0.03	0.3 ± 0.2	0.9 ± 0.2
F3	0.06 ± 0.02	0.1 ± 0.04	1.1 ± 0.3	1.2 ± 0.3
F4	0.1 ± 0.1	0.2 ± 0.3	1.2 ± 0.2	1.2 ± 0.2

**Table 6:** Mean tan δ of Control 2, F3 and F4 at different temperatures.

Formulation	Mean tan δ at 8 °C	Mean tan δ at 25 °C	Mean tan δ at 32 °C	Mean tan δ at 34 °C
Control 2	2.2 ± 0.2	3.3 ± 0.8	0.2 ± 0.01	0.2 ± 0.02
F3	2.5 ± 1.1	3.0 ± 0.2	0.2 ± 0.04	0.1 ± 0.003
F4	1.2 ± 0.4	2.8 ± 1.6	0.1 ± 0.002	0.1 ± 0.01

**Table 7:** Mathematical models for drug release kinetics of F3 and F4.

Formulation	Zero-Order	First-Order	Hixson–Crowell	Korsmeyer–Peppas	
	R^2^	R^2^	R^2^	R^2^	*n*
F3	0.961	0.985	0.981	0.997	0.628
F4	0.956	0.987	0.980	0.998	0.639

**Table 8:** Ex vivo permeation characteristics of clozapine across sheep nasal mucosal tissues (*n* = 3 ± SD).

Formulation	*J_SS_* (μg cm^−2^ h^−1^)	*P_app_* × 10^−3^ (cm h^−1^)	*D* × 10^−4^ (cm^2^ h^−1^)	*C_8_* (μg cm^−2^)
Clozapine solution	139.91 ± 4.01	269.06 ± 7.34	12.50 ± 0.35	851.27 ± 11.02
F3	40.79 ± 2.05 *	74.51 ± 3.74 *	3.46 ± 0.17 *	220.40 ± 7.10 *
F4	32.99 ± 3.01 ^†,^*	60.60 ± 5.53 ^†,^*	2.81 ± 0.26 ^†,^*	186.77 ± 3.01 ^†,^*

* *p* < 0.05 relative to clozapine solution. ^†^ *p* < 0.05 relative to F3.

**Table 9:** Drug stability study of F3 and F4 over 3 months.

Time	0th Month	1st Month	2nd Month	3rd Month
At 8 °C				
F3	1.06 ± 0.002	0.94 ± 0.01	0.93 ± 0.11	0.91 ± 0.01
F4	1.05 ± 0.02	1.14 ± 0.02	0.92 ± 0.15	0.92 ± 0.004
At 25 °C				
F3	1.06 ± 0.06	0.89 ± 0.01	0.83 ± 0.002	0.81 ± 0.01
F4	1.06 ± 0.06	0.87 ± 0.01	0.82 ± 0.004	0.81 ± 0.001
At 40 °C				
F3	1.03 ± 0.03	0.86 ± 0.04	0.87 ± 0.003	0.71 ± 0.002
F4	0.97 ± 0.02	0.87 ± 0.04	0.75 ± 0.001	0.71 ± 0.01
